# Suppressing the Influence of Ectopic Beats by Applying a Physical Threshold-Based Sample Entropy

**DOI:** 10.3390/e22040411

**Published:** 2020-04-04

**Authors:** Lina Zhao, Jianqing Li, Jinle Xiong, Xueyu Liang, Chengyu Liu

**Affiliations:** 1 The State Key Laboratory of Bioelectronics, School of Instrument Science and Engineering, Southeast University, Nanjing 210096, China; zhaolina0808@126.com (L.Z.); 213162269@seu.edu.cn (J.X.); 213163373@seu.edu.cn (X.L.); 2 School of Biomedical Engineering and Informatics, Nanjing Medical University, Nanjing 211166, China

**Keywords:** sample entropy, heart rate variability, ECG, ectopic beat

## Abstract

Sample entropy (SampEn) is widely used for electrocardiogram (ECG) signal analysis to quantify the inherent complexity or regularity of RR interval time series (i.e., heart rate variability (HRV)), with the hypothesis that RR interval time series in pathological conditions output lower SampEn values. However, ectopic beats can significantly influence the entropy values, resulting in difficulty in distinguishing the pathological situation from normal situations. Although a theoretical operation is to exclude the ectopic intervals during HRV analysis, it is not easy to identify all of them in practice, especially for the dynamic ECG signal. Thus, it is important to suppress the influence of ectopic beats on entropy results, i.e., to improve the robustness and stability of entropy measurement for ectopic beats-inserted RR interval time series. In this study, we introduced a physical threshold-based SampEn method, and tested its ability to suppress the influence of ectopic beats for HRV analysis. An experiment on the PhysioNet/MIT RR Interval Databases showed that the SampEn use physical meaning threshold has better performance not only for different data types (normal sinus rhythm (NSR) or congestive heart failure (CHF) recordings), but also for different types of ectopic beat (atrial beats, ventricular beats or both), indicating that using a physical meaning threshold makes SampEn become more consistent and stable.

## 1. Introduction

Entropy is a valuable tool for quantifying the complexity or regularity of cardiovascular time series and provides important insights for understanding the underlying mechanisms of the cardiovascular system. Since the concept of ‘information entropy’ was first proposed by Shannon in 1948 [1], entropy was used as a tool to quantify the quantity of information. Approximate entropy (ApEn) [2], proposed by Pincus et al., is an entropy algorithm initially used in physiological signal analysis as it is adaptive in short-term time series processing. However, ApEn introduces self-matching in calculations, resulting in estimation bias and poor relative consistency [3]. To solve this problem, Richman and Moorman developed an improved version of sample entropy (SampEn) [3], which is based on the calculation of the conditional probability that any two segments of m beats that are similar remain similar when their length increases by one beat. Compared with ApEn, SampEn has a lower estimate bias, better relative consistency and less dependence on data length, which makes it more appropriate in physiological signal processing. SampEn is now the most widely used entropy algorithm in physiological signal analysis.

For entropy calculation, three intrinsic parameters, i.e., the embedding dimension m, the tolerance threshold r and the time series length N need to be initialized. SampEn was reported to not be sensitive to the time series length N if N≥200~300 [4,5]. Parameter m is based on the length N under the suggested relationship of $N\approx 10^{m}\sim 20^{m}$ [6]. Among all three parameters, the tolerance threshold r is the most difficult to be determined. Usually, the recommended r is between 0.10 and 0.25 times the standard deviation (SD) of the physiological data [3,7]. If the r value is too small, the number of matched vectors will be small, and by contrast, if the r value is too big, detailed information within time series will be ignored [8,9]. Moreover, in practice, RR interval time series in different physiological/pathological groups usually have variable SD values, inducing that the comparison between different groups uses different threshold criteria, and it is not easy to find an appropriate r value to achieve an optimal result if simply using the suggested range of 0.10 to 0.25 times the SD.

Researchers have made several useful attempts to improve the performances of entropy measures. One is multiscale analysis. Costa et al. developed a multiscale entropy (MSE) method [10,11], with the hypothesis that MSE can better describe cardiovascular complexity. MSE is based on the evaluation of SampEn in coarse-grained RR interval time series with a coarse-graining order from 1 to a preset scale (such as 10) [12,13]. However, coarse-graining changes the SD of time series and thus changes the corresponding r value [14], resulting in different opinions on the selection of r values, i.e., whether using a fixed tolerance r or using a varying tolerance r adjusted at each scale as a fraction of the SD of the coarse-grained time series is better [10]. Another attempt is the use of fuzzy theory-assisted entropy methods, such as fuzzy entropy developed by Chen et al. [15] and fuzzy measure entropy developed by Liu et al. [16,17], where fuzzy functions are employed to replace the traditional Heaviside function used in SampEn, to improve the statistical stability of SampEn outputs. Herein, although the determination rule for vector similarity is changed, the tolerance r still uses the fixed range of 0.10 to 0.25 times the SD. There are also entropy developments focusing on specific disease detection, such as for the detection of atrial fibrillation (AF) [18,19,20,21], heart failure [22,23], diabetes [24], etc. Specially, Lake and colleagues developed a new AF entropy detector, named the coefficient of sample entropy (COSEn), for AF determination within an extremely short RR interval time series (only 12 RR intervals). COSEn allowed flexibility in choosing the tolerance r and suggested an appropriate choice of a fixed r value of 30 ms [25].

In a previous study, we found that SampEn reported higher values in the normal sinus rhythm (NSR) group than the congestive heart failure (CHF) group when selecting a small threshold r value (*r* = 0.10), but reported lower values when using large threshold r values (*r* = 0.20 or 0.25) [4]. The opposite entropy change trend brings difficulty to defining a unified threshold r to distinguish CHF patients from NSR subjects in heart rate variability (HRV) analysis. To solve this problem, we proposed a physical threshold-based SampEn method to discriminate the opposite entropy change trend in the task of classifying CHF and NSR subjects [26], where the physical threshold-based SampEn was demonstrated to have a better stability than the traditional SampEn.

HRV analysis is based on the analysis of normal RR intervals from the beats generated by the sinoatrial node. Unlike the normal beats generated by the sinoatrial node, ectopic beats are generated by additional electrical impulses imposed by other latent pacemakers [27]. Ectopic beats may cause bias in the reliable measurement of HRV in both the time and frequency domains [28,29], as well as in entropy measurement [30]. Even the presence of only one ectopic beat can introduce an increase in the high frequency power in HRV of around 10% [31]. Although many detection and editing methods for ectopic beats have been proposed [32,33,34], there is no agreed conclusion on how to efficiently remove them. More importantly, the efficiency of editing ectopic beats dramatically decreases when dealing with the dynamic ECG signals due to signal noise. In dynamic ECGs, noises caused by the body’s activities, motion artifacts, electrode interferences etc., are inevitable [35,36]. A recent study demonstrated that even when using state-of-the-art QRS detectors, an 80% or higher accuracy of QRS detection is not achieved. By contrast, these methods can easily obtain a 99% accuracy using conventional ECG databases such as the PhysioNet/MIT Arrythmias database [37]. Potential detection errors from the automatic analysis of dynamic ECGs also bring abnormal RR intervals, i.e., RR intervals lasting for too much or too little time. The existence of either the ectopic beats or the falsely detected QRS locations can significantly contaminate the entropy outputs.

Thus, the effectiveness of entropy measures, typically SampEn, should be re-checked for analyzing the dynamic ECG signals. A predictable situation is that SampEn may change a lot if moving the analysis window from an ectopic-free RR interval time series to an entopic one. Thus, it is necessary to further develop an entropy method, which can keep relatively stable when randomly dealing with the ectopic or ectopic-free RR interval time series for a specific subject/patient. Due to the fact that it is difficult to identify the abnormal RR intervals caused by noises or true ectopic beats in the automatic analysis for dynamic ECGs, this necessity becomes urgent and practical for real signal processing. In this study, we aimed to test the performance of a new physical threshold-based SampEn when applied to RR interval time series with ectopic beats, to explore if it can efficiently suppress the sudden change in entropy results due to the appearance of ectopic beats, i.e., to verify its ability to suppress the influence of ectopic beats for HRV analysis.

## 2. Methods

### 2.1. Data

All data used were from the PhysioNet/MIT RR Interval Databases from http://www.physionet.org [38], a free-access, online archive of physiological signals. The NSR RR Interval database includes 54 long-term RR interval recordings of subjects with normal sinus rhythms aged from 29 to 76. The CHF RR Interval database includes 29 long-term RR interval recordings of subjects aged from 34 to 79, with CHF diagnoses (NYHA classes I, II and III). Each of the long-term RR interval recordings is a 24-h recording, including both day-time and night-time. Both the NSR and CHF subjects took the Holter ECG measurement under a similar level of physical activity. The original ECG signals were digitized at 128 Hz, and the beat annotations were obtained by automated analysis with manual review and correction.

**Figure 1:**
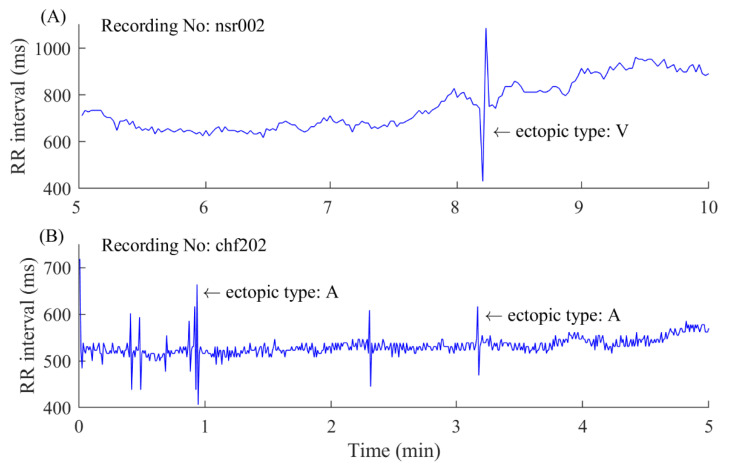
Examples of 5-min ectopic RR segments. (**A**) An ectopic segment with ventricular (V) ectopic beats from an normal sinus rhythm (NSR) subject. (**B**) An ectopic segment with atrial (A) ectopic beats from a congestive heart failure (CHF) patient. Please note there are other atrial ectopic beats in this 5-min RR segment, where the RR interval values have sudden changes.

A 5-min time window was used to segment the long-term RR interval records. The 5-min RR segments with at least one ectopic beat were extracted as ectopic segments used in this study. Information regarding ectopic beats was manually annotated by experts and was given in the database, classifying them into two types: atrial (A) or ventricular (V) beats, depending on the localization of the ectopic focus. In each 5-min RR segment, RR intervals greater than 2 s, but not ectopic intervals, were removed, since they are all noisy intervals arising from artificial influences [4]. Figure 1 shows examples of ectopic RR segments from an NSR subject and a CHF patient. Table 1 and Table 2 summarize the numbers of ectopic beats and ectopic 5-min segments in each of the 54 NSR and 29 CHF records. For each recording (subject), we only chose the recordings with more than 10 ectopic segments, while excluding the ectopic segments with more than 6 ectopic beats, since the majority of ectopic segments have 1–5 ectopic beats.

### 2.2. Physical Threshold-Based SampEn

The calculation process for the physical threshold-based SampEn is summarized as follows [26]:

For the RR segment x(i) (1≤i≤N), given the parameters m and r, first formed is the vector sequence $X_{i}^{m}$:

(1) $$X_{i}^{m}=\left\{x\left(i\right),\;x\left(i+1\right),\cdots ,x\left(i+m-1\right)\right\}\;1\leq i\leq N-m$$

The vector $X_{i}^{m}$ represents m consecutive x(i) values. Then, the distance between $X_{i}^{m}$ and $X_{j}^{m}$ based on the maximum absolute difference is defined as:

(2) $$d_{i,j}^{m}=d\left[X_{i}^{m},X_{j}^{m}\right]=\underset{0\leq k\leq m-1}{\max}\left|x\left(i+k\right)-x\left(j+k\right)\right|$$

For each $X_{i}^{m},$ denote $B_{i}^{m}\left(r\right)$ as ${\left(N-m\right)}^{-1}$ times the number of $X_{j}^{m}$ (1≤j≤N−m) that meets $d_{i,j}^{m}\leq r$. Similarly, set $A_{i}^{m}\left(r\right)$ is ${\left(N-m\right)}^{-1}$ times the number of $X_{j}^{m+1}$ that meets $d_{i,j}^{m+1}\leq r$ for all 1≤j≤N−m. Instead of using the traditional threshold, which is between 0.10 and 0.25 times the SD of the data, herein, a physical threshold r is used to form a unified comparison baseline for determining the vector similarity. As the raw ECG signals were digitized at 128 Hz, which means that the difference between any two vectors is approximately an integer multiple of 8 ms, here we used r=12 ms as the physical threshold according to the previous suggestion [10].

Then, SampEn is defined by:

(3) $$\mathrm{SampEn}\left(m,r,N\right)=-\ln\left(\overset{N-m}{\underset{i=1}{\sum }}A_{i}^{m}\left(r\right)/\overset{N-m}{\underset{i=1}{\sum }}B_{i}^{m}\left(r\right)\right)$$

In addition, previous studies suggested that using an embedding dimension of m=1 or 2 can obtain better results for classifying NSR and CHF groups when setting the RR time series length as N=300 [4]. In this study, we kept this suggestion of m=1 and 2.

To test the performance of physical threshold-based SampEn, traditional SampEn was used as the comparative method. Entropy values were first calculated from the raw ectopic 5-min RR segments. Then, the ectopic RR intervals in these ectopic RR segments were removed to form the ectopic-free RR segments. Finally, entropy values were re-calculated from these constructed ectopic-free RR segments. Entropy variances before and after ectopic beat removal were calculated, and the variation could be regarded as an index for evaluating the performance of entropy measures’ abilities to suppress the influence of ectopic beats.

## 3. Results

### 3.1. Demonstration of the Influence of Ectopic Beats on Entropy Values

**Figure 2:**
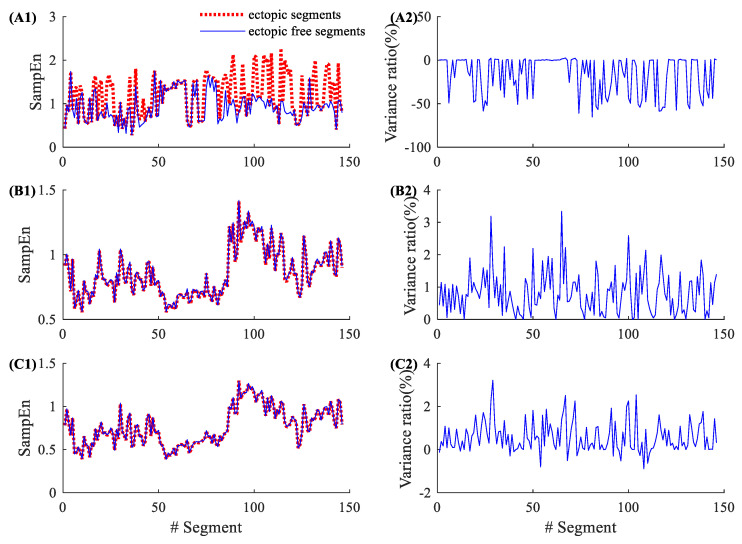
An example of the influence of ectopic beats. Entropy values and their variance ratios for subject NSR002 before and after the ectopic beat removal: (**A1**) entropy results and (**A2**) their variance ratios for the traditional SampEn (m=2, r=0.2), (**B1**) entropy results and (**B2**) their variance ratios for the physical threshold-based SampEn (m=1, r=12 ms), and (**C1**) entropy results and (**C2**) their variance ratios for the physical threshold-based SampEn (m=2, r=12 ms).

Figure 2 shows the entropy results from an NSR subject (NSR002). As shown in Table 1, NSR002 has a total of 146 5-min ectopic RR segments. The left panels in Figure 2 show the entropy values for these 146 ectopic RR segments before ectopic RR interval removal (red dotted line) and after ectopic RR interval removal (blue line). The traditional SampEn has a large variation before and after ectopic RR interval removal, while the new physical threshold-based SampEn has very small changes when analyzing ectopic free segments. The right panels show the corresponding variance ratios, i.e., the entropy value of the ectopic free segment minus the entropy value of ectopic segment, divided by the entropy value of the ectopic segment. The entropy variance ratios in SampEn varied from −65.24% to 2.25%, with an average of −16.32% and an SD of 21.93%. The corresponding variance ratios for the physical threshold-based SampEn varied from 0% to 3.34% (m=1, r=12 ms), with an average of 0.81% and an SD of 0.66%; and from −0.51% to 3.21% (m=2, r=12 ms), with an average of 0.57% and an SD of 0.72%. Compared with the traditional SampEn, the physical threshold-based SampEn showed significantly lower variance ratios, demonstrating the better robustness of the new SampEn method.

**Figure 3:**
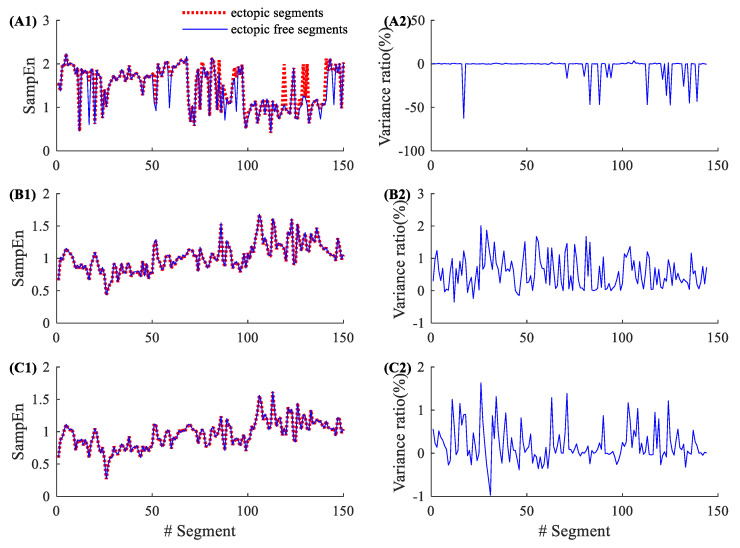
An example of the influence of ectopic beats. Entropy values and their variance ratios for subject CHF202 before and after the ectopic beat removal: (**A1**) entropy results and (**A2**) their variance ratios for the traditional SampEn (m=2, r=0.2), (**B1**) entropy results and (**B2**) their variance ratios for the physical threshold-based SampEn (m=1, r=12 ms), and (**C1**) entropy results and (**C2**) their variance ratios for the physical threshold-based SampEn (m=2, r=12 ms).

By contrast, Figure 3 shows similar results from a CHF patient (CHF202), which has a total of 150 ectopic RR segments, as shown in Table 2. The entropy variance ratios in SampEn varied from −62.50% to 3.53%, with an average of −3.18% and an SD of 11.36%. The corresponding variance ratios for physical threshold-based SampEn varied from −0.35% to 2.01% (m=1, r=12 ms), with an average of 0.55% and an SD of 0.49%; and from −0.98% to 1.39% (m=2, r=12 ms), with an average of 0.20% and an SD of 0.42%. Compared with the traditional SampEn, the physical threshold-based SampEn also showed significantly lower variance ratios in the demonstrated CHF patient.

### 3.2. Demonstration of the Influence of Atrial Beats on Entropy Values

There are two types of ectopic beat in the used PhysioNet/MIT RR Interval Databases, atrial and ventricular beats (shown in Figure 1). To further test the robustness of physical threshold-based SampEn method, we analyzed the ectopic segments only containing atrial or ventricular beats. For NSR002, there are 17 segments containing atrial beats and 137 segments containing ventricular beats among all 146 ectopic RR segments. For CHF202, there are 41 segments containing atrial beats and 123 segments containing ventricular beats among all 150 ectopic RR segments.

**Figure 4:**
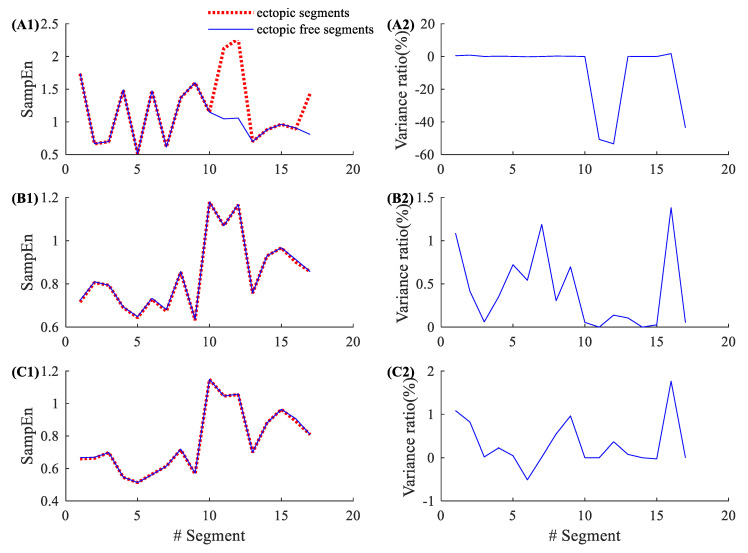
An example of the influence of atrial ectopic beats. Entropy values and their variance ratios for subject NSR002 (only 17 atrial ectopic RR segments) before and after the ectopic beat removal: (**A1**) entropy results and (**A2**) their variance ratios for the traditional SampEn (m=2, r=0.2), (**B1**) entropy results and (**B2**) their variance ratios for the physical threshold-based SampEn (m=1, r=12 ms), and (**C1**) entropy results and (**C2**) their variance ratios for the physical threshold-based SampEn (m=2, r=12 ms).

**Figure 5:**
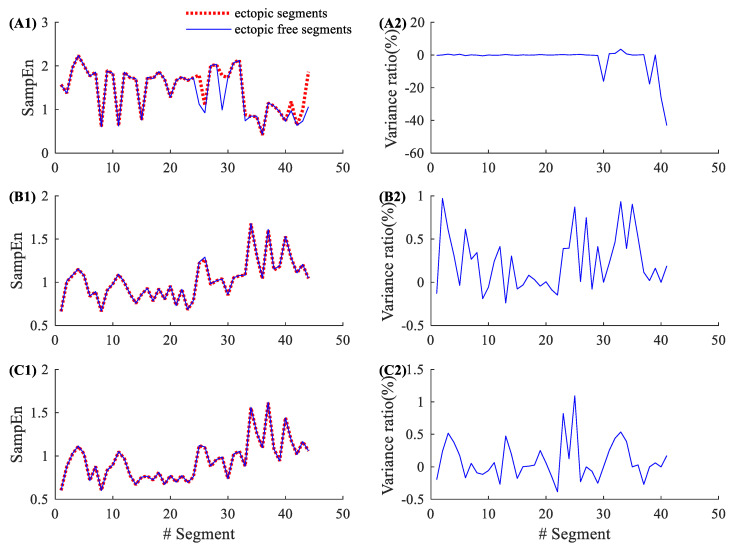
An example of the influence of atrial ectopic beats. Entropy values and their variance ratios on subject CHF202 (only 41 atrial ectopic RR segments) before and after the ectopic beat removal: (**A1**) entropy results and (**A2**) their variance ratios for the traditional SampEn (m=2, r=0.2), (**B1**) entropy results and (**B2**) their variance ratios for the physical threshold-based SampEn (m=1, r=12 ms), and (**C1**) entropy results and (**C2**) their variance ratios for the physical threshold-based SampEn (m=2, r=12 ms).

Figure 4 shows the results of 17 atrial ectopic RR segments from NSR002. Entropy variance ratios in SampEn varied from −53.40% to 1.77%, with an average of −8.48% and an SD of 19.54%. The corresponding variance ratios for physical threshold-based SampEn varied from 0% to 1.38% (m=1, r=12 ms), with an average of 0.42% and an SD of 0.45%; and from −0.51% to 1.77% (m=2, r=12 ms), with an average of 0.32% and an SD of 0.56%. Compared with the traditional SampEn, the physical threshold-based SampEn showed significantly lower variance ratios for the analysis of atrial ectopic RR segments. Figure 5 shows the similar results from CHF202, which includes 41 atrial ectopic RR segments. The entropy variance ratios in the SampEn varied from −43.10% to 3.53%, with an average of −2.34% and an SD of 8.51%. The corresponding variance ratios for physical threshold-based SampEn varied from −0.19% to 0.97% (m=1, r=12 ms), with an average of 0.24% and an SD of 0.33%; and from −0.39% to 1.09% (m=2, r=12 ms), with an average of 0.10% and an SD of 0.30%. The results for CHF also support that the physical threshold-based SampEn had significantly lower variance ratios in the analysis of atrial ectopic RR segments.

### 3.3. Demonstration of the Influence of Ventricular Beats on Entropy Values

**Figure 6:**
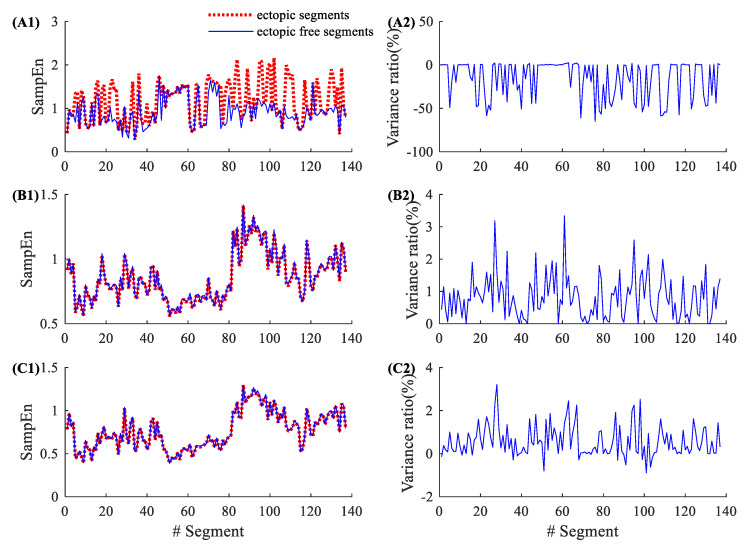
An example of the influence of ventricular ectopic beats. Entropy values and their variance ratios for subject NSR002 (only 137 ventricular ectopic RR segments) before and after the ectopic beat removal: (**A1**) entropy results and (**A2**) their variance ratios for the traditional SampEn (m=2, r=0.2), (**B1**) entropy results and (**B2**) their variance ratios for the physical threshold-based SampEn (m=1, r=12 ms), and (**C1**) entropy results and (**C2**) their variance ratios for the physical threshold-based SampEn (m=2, r=12 ms).

**Figure 7:**
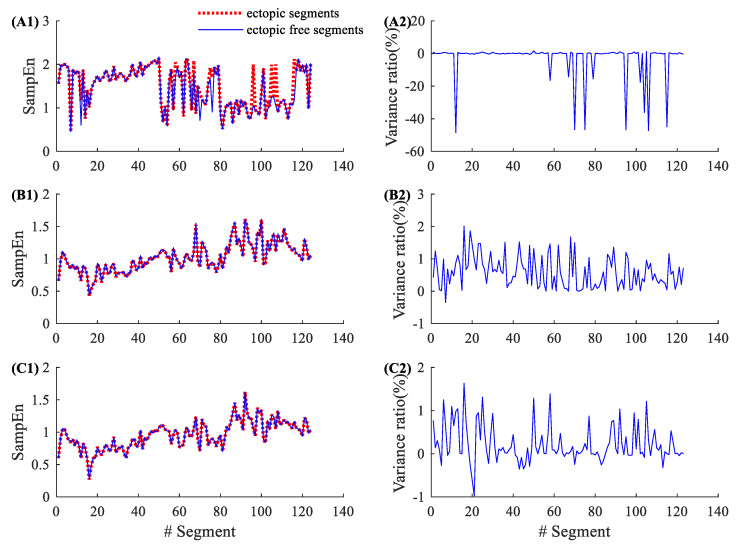
An example of the influence of ventricular ectopic beats. Entropy values and their variance ratios for subject CHF202 (only 123 ventricular ectopic RR segments) before and after the ectopic beat removal: (**A1**) entropy results and (**A2**) their variance ratios for the traditional SampEn (m=2, r=0.2), (**B1**) entropy results and (**B2**) their variance ratios for the physical threshold-based SampEn (m=1, r=12 ms), and (**C1**) entropy results and (**C2**) their variance ratios for the physical threshold-based SampEn (m=2, r=12 ms).

Figure 6 shows the results of 137 ventricular ectopic RR segments from NSR002. Entropy variance ratios in SampEn varied from −65.24% to 2.46%, with an average of −16.15% and an SD of 21.57%. The corresponding variance ratios for physical threshold-based SampEn varied from 0% to 3.34% (m=1, r=12 ms), with an average of 0.82% and an SD of 0.66%; and from −0.89% to 3.22% (m=2, r=12 ms), with an average of 0.57% and an SD of 0.73%. Compared with the traditional SampEn, the physical threshold-based SampEn also showed significantly lower variance ratios in the analysis of ventricular ectopic RR segments. Figure 7 shows the similar results from CHF202, which includes 123 ventricular ectopic RR segments. The entropy variance ratios in SampEn varied from −48.55% to 1.56%, with an average of −2.97% and an SD of 10.89%. The corresponding variance ratios for the physical threshold-based SampEn varied from −0.35% to 2.01% (m=1, r=12 ms), with an average of 0.59% and an SD of 0.49%; and varied from −0.98% to 1.63% (m=2, r=12 ms), with an average of 0.22% and an SD of 0.43%. The results for CHF also support the idea that the physical threshold-based SampEn had lower variance ratios in the analysis of ventricular ectopic RR segments.

### 3.4. Total Results

**Figure 8:**
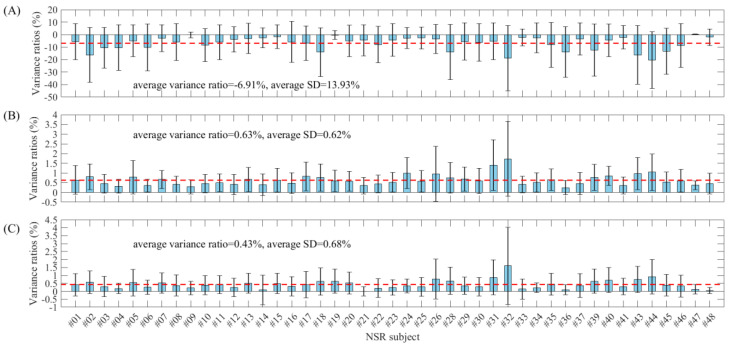
Box plots of the entropy variance ratios and standard deviations for each subject in the NSR group. (**A**) Traditional SampEn (m=2, r=0.2), (**B**) physical threshold-based SampEn (m=1, r=12 ms) and (**C**) physical threshold-based SampEn (m=2, r=12 ms).

Table 3 and Figure 8 show the entropy variance ratios and standard deviations for each subject in the NSR group (in total, 45 recordings with the required numbers of ectopic segments, as indicated in Table 1) when comparing the entropy values from both before and after ectopic beat removal. The absolute variance ratio and standard deviation of SampEn for each subject were obviously larger than those from the two physical threshold-based SampEn methods, and the mean variance ratios were −6.91%, 0.63% and 0.43% for SampEn and the two physical threshold-based SampEn methods (m=1 and m=2 respectively, and, for both, r=12 ms). In addition, SampEn showed significantly larger standard deviations of entropy variance ratios within subjects than the two physical threshold-based SampEn methods. The average standard deviations were 13.93%, 0.62% and 0.68% for SampEn and the two physical threshold-based SampEn methods (m=1 and m=2 respectively, and, for both, r=12 ms).

**Figure 9:**
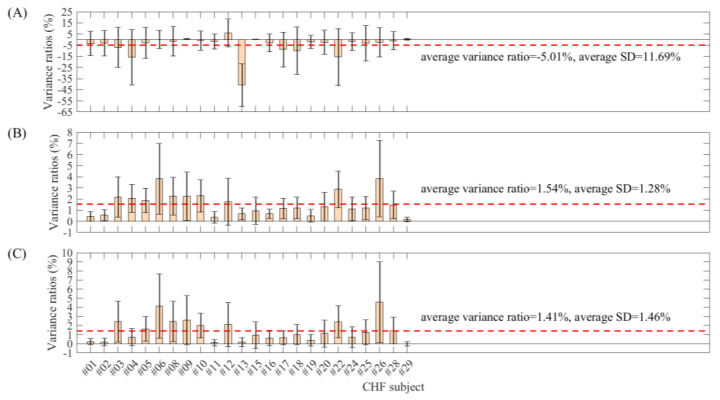
Box plots of the entropy variance ratios and standard deviations for each subject in the CHF group. (**A**) Traditional SampEn (m=2, r=0.2), (**B**) physical threshold-based SampEn (m=1, r=12 ms) and (**C**) physical threshold-based SampEn (m=2, r=12 ms).

Similarly, Table 4 and Figure 9 show the entropy variance ratios and standard deviations for each patient in the CHF group (24 recordings). The absolute variance ratio and standard deviation for each subject of SampEn were obviously larger than those from the two physical threshold-based SampEn methods, and the mean variance ratios were −5.01%, 1.54% and 1.41% for SampEn and the two physical threshold-based SampEn methods (m=1 and m=2 respectively, and, for both, r=12 ms). Meanwhile, SampEn showed significantly larger standard deviations of entropy variance ratios within patients than the two physical threshold-based SampEn methods. The average standard deviations were 11.69%, 1.28% and 1.46% for SampEn and the two physical threshold-based SampEn methods (m=1 and m=2 respectively, and, for both, r=12 ms). These results further confirmed the better stability of SampEn using the physical threshold.

When comparing the group differences of variance ratios between the NSR and CHF groups, the traditional SampEn showed no significant difference (*P* = 0.3) while the physical threshold-based SampEn showed significant differences (both *P* < 0.01 for two parameter m settings), with *P* = 4 ×10^−7^ for m=1 and *P* = 2 ×10^−6^ for m=2 respectively.

## 4. Discussion and Conclusions

In all of the three intrinsic parameters of SampEn, the parameter r is the most difficult to be determined. Different opinions regarding the selection of threshold r would lead to different entropy outputs. In a previous study, researchers developed different methods for the selection of the threshold r [8,39], and tried to make the selection method more rigorous and standardized [4,40]. However, there is no unified standard for r value selection now. Special selection methods only perform well under specific circumstances, and the influencial factors may include data type, data length, disease type, etc. Therefore, the argument has always been whether to use a fixed tolerance r or a varying tolerance r. Researchers first explored this issue in the MSE method, which performed SampEn analysis on several different scales and thus induced the question of whether using a fixed or a varying tolerance r at different scales was better. Angelini et al. reported that using a fixed and a varying tolerance r in MSE generated similar changes in CHF analysis [41]. Silva et al. also confirmed this finding in a rat model of hypertension and CHF [42], suggesting that the selection of the tolerance r in the MSE method is not relevant. However, the fixed tolerance r at different scales only stays the same for special subjects. For different subjects, there is also an inter-variability of the tolerance r*,* since different subjects have different signal variabilities of time series.

In a previous study, we found that SampEn reported lower values in CHF patients when using a small threshold r value (r=0.10), but higher values when using large threshold r values (r=0.20 or 0.25). The opposite entropy change trend brings difficulty to the clinical explanation. To solve this problem, we proposed a physical threshold-based SampEn method to discriminate the opposite entropy change trend in classifying CHF and NSR subjects. This previous study was performed only on RR segments without any ectopic beats. The raw ECG signal had a sample rate of 128 Hz, generating differences of roughly 8 ms and its multiples for RR intervals. Thus, we tested the effects of different r values of r=12 ms, r=20 ms, r=28 ms etc., and found that r=12 ms provided the best discrimination between the CHF and NSR groups. In this study, we used the previously proposed fixed tolerance r method with r=12 ms [26] with physical meaning to analyze the RR interval time series with ectopic beats, to explore if the new r method has better performance for ectopic time series. Forty-five NSR and 24 CHF recordings were enrolled in this study, all of which had an appreciable number of ectopic beats, including atrial and ventricular beats. SampEn entropy results from both the traditional varying threshold (a fraction of the SD of time series) and the new fixed physical meaning threshold were compared before and after ectopic beat removal. For both the NSR and CHF groups, the entropy variance of SampEn with the traditional threshold is obviously larger than that when using the physical meaning threshold, which verifies the better consistency of the new physical meaning threshold method.

Ectopic beats are routinely removed or edited from the RR interval time series prior to HRV analysis. Salo et al. found that both time- and frequency-domain indices were sensitive to the editing of RR intervals [28]. This finding was consistent with our current study, where we showed that the SampEn calculated by the traditional method was sensitive to the removal of ectopic beats (one to five beats). The reason is that the ectopic beats usually result in sudden changes in the RR interval time series. This effect is significant on the transient change of HRV reflected by both the time- and frequency-domain indices, as well as nonlinear indices like SampEn [29,43]. However, for each subject, after ectopic beats were removed, the entropy value only changed significantly in specific segments. The entropy value variance for all segments in subject NSR002 was between −65.24% and 2.25% for the traditional threshold; and between 0% and 3.34% (m=1), and −0.51% and 3.21% (m=2) for the two physical meaning thresholds. The results in subject CHF202 were similar, i.e., between −62.50% and 3.53% for the traditional threshold; and −0.35% and 2.01% (m=1), and −0.98% and 1.39% (m=2) for the two physical meaning thresholds. The absolute change in SampEn with the traditional threshold was much more significant than that in SampEn with the physical meaning threshold.

In addition, we also analyzed the effect of different ectopic beats (atrial or ventricular) on the tested SampEn output. Results from the segments only containing atrial or ventricular beats showed that SampEn using the physical meaning threshold still performed better than SampEn using the traditional threshold. When atrial beats or ventricular beats were removed, the absolute entropy value variation in the former SampEn was significantly smaller than that in the latter.

In conclusion, SampEn using the physical meaning threshold has better performance, not only for different data types (NSR or CHF recordings), but also for different types of ectopic beat (atrial beats, ventricular beats, or both), and using the physical meaning threshold makes SampEn become more consistent and stable.

## Figures and Tables

**Table 1:** A summary of the ectopic beats and segments in the PhysioNet/MIT RR Interval Databases for the NSR group.

Record	# Ectopic Beats	# Ectopic Segments	Record	# Ectopic Beats	# Ectopic Segments
NSR001	81	58	NSR028	166	95
NSR002	233	146	NSR029	24	18
NSR003	50	37	NSR030	92	58
NSR004	36	33	NSR031	630	191
NSR005	611	198	NSR032	490	188
NSR006	96	40	NSR033	15	14
NSR007	113	81	NSR034	21	18
NSR008	70	50	NSR035	43	29
NSR009	30	25	NSR036	169	28
NSR010	206	107	NSR037	31	29
NSR011	152	92	NSR038 *	6	4
NSR012	46	40	NSR039	131	87
NSR013	38	32	NSR040	40	17
NSR014	305	112	NSR041	32	29
NSR015	36	24	NSR042 *	11	10
NSR016	47	42	NSR043	241	123
NSR017	958	265	NSR044	5225	270
NSR018	547	213	NSR045	233	149
NSR019	42	33	NSR046	302	94
NSR020	169	108	NSR047	22	22
NSR021	12	12	NSR048	31	21
NSR022	56	47	NSR049 *	3	3
NSR023	53	34	NSR050 *	3	3
NSR024	8033	272	NSR051 *	6	6
NSR025	492	120	NSR052 *	13	10
NSR026	92	44	NSR053 *	1	1
NSR027 *	5	5	NSR054 *	9	8

* indicates the recordings excluded for the analysis since there are no 10 or more ectopic 5-min RR segments including 5 or fewer ectopic beats.

**Table 2:** A summary of the ectopic beats and segments in the PhysioNet/MIT RR Interval Databases for the CHF group.

Record	# Ectopic Beats	# Ectopic Segments	Record	# Ectopic Beats	# Ectopic Segments
CHF201	61	36	CHF216	18	14
CHF202	273	150	CHF217	779	228
CHF203	496	187	CHF218	2667	217
CHF204	2297	247	CHF219	37	28
CHF205	1356	245	CHF220	820	143
CHF206	11,112	240	CHF221 *	11,608	276
CHF207 *	15,189	249	CHF222	2792	274
CHF208	3073	257	CHF223 *	5410	274
CHF209	507	156	CHF224	356	150
CHF210	2122	258	CHF225	242	121
CHF211	14	11	CHF226	1638	257
CHF212	3483	205	CHF227 *	5649	275
CHF213	10,968	281	CHF228	1467	204
CHF214 *	21,160	204	CHF229	22	20
CHF215	5851	166			

* indicates the recordings excluded for the analysis since there are no 10 or more ectopic 5-min RR segments including 5 or fewer ectopic beats.

**Table 3:** Entropy variance ratios and standard deviations for each subject in the NSR group.

Record	Variance Ratios (%)			Standard Deviation (%)		
	*m* = 2, *r* = 0.2	*m* = 1, *r* = 12 ms	*m* = 2, *r* = 12 ms	*m* = 2, *r* = 0.2	*m* = 1, *r* = 12 ms	*m* = 2, *r* = 12 ms
NSR001	−5.62	0.64	0.41	14.43	0.74	0.69
NSR002	−16.32	0.81	0.57	21.93	0.66	0.72
NSR003	−10.60	0.45	0.30	16.37	0.49	0.64
NSR004	−10.41	0.32	0.18	18.24	0.35	0.33
NSR005	−4.91	0.79	0.55	12.72	0.86	0.83
NSR006	−10.24	0.35	0.26	18.68	0.33	0.44
NSR007	−2.81	0.67	0.53	10.74	0.46	0.64
NSR008	−6.07	0.42	0.37	14.75	0.42	0.66
NSR009	−0.17	0.29	0.21	2.27	0.36	0.42
NSR010	−8.40	0.46	0.38	13.15	0.48	0.60
NSR011	−6.05	0.50	0.43	14.07	0.45	0.57
NSR012	−3.70	0.41	0.24	10.15	0.52	0.58
NSR013	−3.13	0.67	0.51	12.16	0.62	0.62
NSR014	−2.55	0.40	0.09	8.06	0.55	0.93
NSR015	−1.66	0.63	0.49	9.53	0.61	0.64
NSR016	−5.86	0.48	0.32	16.40	0.53	0.60
NSR017	−6.81	0.83	0.42	13.86	0.74	0.84
NSR018	−14.06	0.77	0.62	19.45	0.70	0.85
NSR019	−0.31	0.60	0.64	3.52	0.55	0.75
NSR020	−5.11	0.58	0.52	12.63	0.51	0.68
NSR021	−4.51	0.35	0.00	12.49	0.42	0.28
NSR022	−7.99	0.44	0.20	14.61	0.46	0.59
NSR023	−4.27	0.52	0.24	13.01	0.50	0.48
NSR024	−2.79	1.00	0.34	8.37	0.79	0.44
NSR025	−2.64	0.57	0.28	8.69	0.55	0.60
NSR026	−3.59	0.96	0.77	11.62	1.42	1.25
NSR028	−13.87	0.76	0.66	22.18	0.79	0.85
NSR029	−5.62	0.69	0.35	14.90	0.62	0.55
NSR030	−6.30	0.60	0.29	15.06	0.65	0.57
NSR031	−5.44	1.40	0.88	14.75	1.30	1.10
NSR032	−18.85	1.73	1.61	25.99	1.92	2.43
NSR033	−2.27	0.41	0.14	6.77	0.42	0.64
NSR034	−2.58	0.52	0.22	11.91	0.49	0.30
NSR035	−8.24	0.66	0.45	17.83	0.55	0.69
NSR036	−13.94	0.25	0.10	20.16	0.36	0.31
NSR037	−3.47	0.46	0.37	12.94	0.57	0.75
NSR039	−12.35	0.78	0.64	20.96	0.67	0.77
NSR040	−4.44	0.85	0.71	13.05	0.49	0.78
NSR041	−2.20	0.36	0.30	9.36	0.43	0.52
NSR043	−16.28	0.97	0.74	23.58	0.82	0.82
NSR044	−20.46	1.04	0.92	22.74	0.93	1.09
NSR045	−13.36	0.53	0.39	18.43	0.52	0.67
NSR046	−8.72	0.60	0.33	17.58	0.58	0.69
NSR047	0.18	0.37	0.13	0.35	0.23	0.30
NSR048	−2.10	0.46	0.05	6.53	0.54	0.19
Average	−6.91	0.63	0.43	13.93	0.62	0.68

**Table 4:** Entropy variance ratios and standard deviations for each subject in the CHF group

Record	Variance Ratios (%)			Standard Deviation (%)		
	*m* = 2, *r* = 0.2	*m* = 1, *r* = 12 ms	*m* = 2, *r* = 12 ms	*m* = 2, *r* = 0.2	*m* = 1, *r* = 12 ms	*m* = 2, *r* = 12 ms
CHF201	−3.48	0.44	0.22	10.98	0.44	0.33
CHF202	−3.18	0.55	0.20	11.36	0.49	0.42
CHF203	−7.00	2.19	2.43	18.09	1.82	2.24
CHF204	−15.87	2.06	0.73	24.74	1.25	0.94
CHF205	−2.81	1.88	1.64	13.97	1.09	1.36
CHF206	0.09	3.82	4.14	8.16	3.17	3.53
CHF208	−1.49	2.27	2.45	13.20	1.70	2.21
CHF209	0.45	2.26	2.59	0.61	2.16	2.70
CHF210	−0.85	2.28	2.01	8.64	1.45	1.35
CHF211	−1.74	0.35	0.11	6.69	0.51	0.36
CHF212	6.02	1.76	2.10	12.43	2.11	2.41
CHF213	−40.79	0.66	0.19	19.12	0.54	0.49
CHF215	0.20	0.96	0.95	0.28	1.21	1.44
CHF216	−2.91	0.68	0.63	7.90	0.43	0.84
CHF217	−8.92	1.15	0.66	15.62	0.91	0.79
CHF218	−9.88	1.21	1.00	21.18	0.95	1.10
CHF219	−2.05	0.50	0.38	5.81	0.57	0.63
CHF220	−2.24	1.30	1.13	10.77	1.30	1.46
CHF222	−15.62	2.87	2.41	25.51	1.64	1.76
CHF224	−1.79	1.12	0.72	7.89	1.06	1.13
CHF225	−3.22	1.19	1.26	15.88	1.03	1.38
CHF226	−2.36	3.85	4.56	13.11	3.43	4.44
CHF228 CHF201	−1.06	1.48	1.44	7.95	1.24	1.47
CHF229	0.24	0.17	−0.01	0.70	0.21	0.24
Average	−5.01	1.54	1.41	11.69	1.28	1.46
